# Vertebral Endplate Concavity in Lateral Lumbar Interbody Fusion: Tapered 3D-Printed Porous Titanium Cage versus Squared PEEK Cage

**DOI:** 10.3390/medicina59020372

**Published:** 2023-02-15

**Authors:** Naoki Segi, Hiroaki Nakashima, Ryuichi Shinjo, Yujiro Kagami, Masaaki Machino, Sadayuki Ito, Jun Ouchida, Kazuaki Morishita, Ryotaro Oishi, Ippei Yamauchi, Shiro Imagama

**Affiliations:** 1 Department of Orthopedic Surgery, Graduate School of Medicine, Nagoya University, 65 Tsurumai, Showa-ku, Nagoya 466-8560, Japan; 2 Department of Orthopedic Surgery, Anjo Kosei Hospital, 28 Higashihirokute, Anjo 446-8602, Japan

**Keywords:** cage subsidence, extreme lateral interbody fusion, lateral lumber interbody fusion, poly-ether-ether-ketone, three-dimensional porous titanium, vertebral endplate injury

## Abstract

*Background and Objectives*: To prevent postoperative problems in extreme lateral interbody fusion (XLIF), it is critical that the vertebral endplate not be injured. Unintentional endplate injuries may depend on the cage. A novel porous titanium cage for XLIF has improved geometry with a tapered tip and smooth surface. We hypothesized that this new cage should lead to fewer endplate injuries. *Materials and Methods*: This retrospective study included 32 patients (mean 74.1 ± 6.7 years, 22 females) who underwent anterior and posterior combined surgery with XLIF for lumbar degenerative disease or adult spinal deformity from January 2018 to June 2022. A tapered 3D porous titanium cage (3DTi; 11 patients) and a squared PEEK cage (sPEEK; 21 patients) were used. Spinal alignment values were measured on X-ray images. Vertebral endplate concavity (VEC) was defined as concavity ≥ 1 mm of the endplate on computed tomography (CT) images, which were evaluated preoperatively and at 1 week and 3 months postoperatively. *Results*: There were no significant differences in the patient demographic data and preoperative and 3-month postoperative spinal alignments between the groups. A 3DTi was used for 25 levels and an sPEEK was used for 38 levels. Preoperative local lordotic angles were 4.3° for 3DTi vs. 4.7° for sPEEK (*p* = 0.90), which were corrected to 12.3° and 9.1° (*p* = 0.029), respectively. At 3 months postoperatively, the angles were 11.6° for 3DTi and 8.2° for sPEEK (*p* = 0.013). VEC was present in 2 levels (8.0%) for 3DTi vs. 17 levels (45%) for sPEEK (*p* = 0.002). After 3 months postoperatively, none of the 3DTi had VEC progression; however, eight (21%) levels in sPEEK showed VEC progression (*p* = 0.019). *Conclusions*: The novel 3DTi cage reduced endplate injuries by reducing the endplate load during cage insertion.

## 1. Introduction

**Figure 1:**
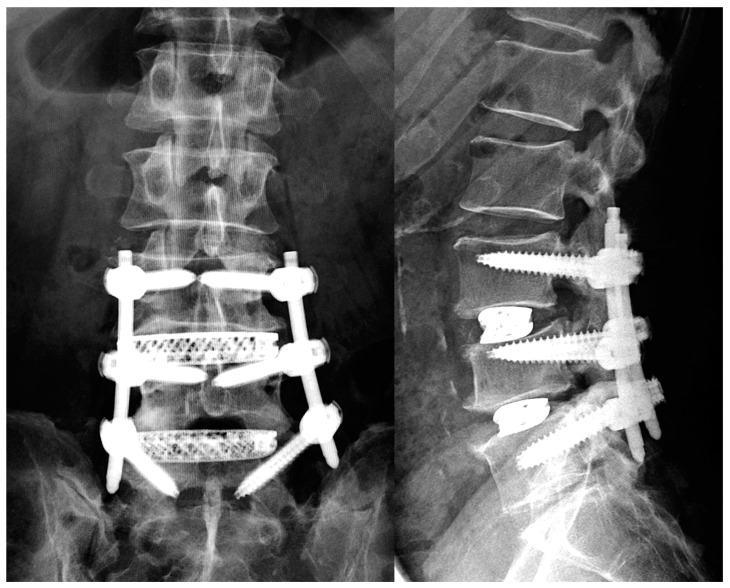
Lumbar degenerative spondylolisthesis treated by 3DTi cage and PPS.

Extreme lateral interbody fusion (XLIF) [1] has become widely used because of its strong corrective force and minimally invasive nature (Figure 1). The corrective force of XLIF is driven by the scaffolding of the cage on the sturdy portion of the vertebral endplate, which depends on the support of the undamaged vertebral endplate. An important complication of XLIF is cage subsidence, which can lead to clinical problems, such as loss of disc height compensation, loss of spinal alignment, recurrent pain, and vertebral fractures [2]. Once the endplates are injured, the correction force of the cage is at least partially lost, and the patient is at risk of further subsequent subsidence and the worsening of clinical improvement [3]. Therefore, it is critical that the vertebral endplate not be injured during XLIF cage insertion.

Unintentional intraoperative endplate injuries in XLIF have been identified in 10.4% to 20.4% of cases [4,5]. The vertebral endplate injuries were not correlated with surgical experience but rather with patient factors, such as older age, female gender, low bone density, preoperative disc angle, and vertebral endplate stiffening, as well as cage height [2,4,5]. During cage insertion, the vertebral endplate is subjected to a load, which depends on the difference between the void space size, the cage to be inserted, and the difference in the elastic moduli between the vertebral bone and cage, as well as on the surface properties of the cage (and associated frictional forces) and the cage geometry (the presence of sharp edges that would cause increased local stresses). Although some of these parameters have been identified in the laboratory [6], it is unclear what effect the cage material and shape has in patients.

A novel cage [7] recently introduced for XLIF is made of porous titanium using three-dimensional (3D)-printing technology and has improved geometry, with tapered tips and smooth surfaces that eliminate spikes. These improvements are expected to reduce endplate loading during insertion. According to previous reports, porous titanium can reduce the risk of cage subsidence and create biostability through bone ongrowth and ingrowth [8]. However, we determined the superiority of this new cage from a different perspective.

The first report for this cage showed a low subsidence rate [7]. In addition, at 6–12 months after standalone XLIF surgery, subsidence was significantly lower for the 3D-printed porous titanium (3DTi) cage than for a poly-ether-ether-ketone (PEEK) cage [9,10]. Furthermore, a report suggested early bone fusion with the 3DTi cage [11]. However, all of these reports showed the results of bone ongrowth and bone remodeling that occurred over time after surgery. In addition to these superiorities of this new cage, our preliminary observations suggested that the new 3DTi cages reduce the postoperative vertebral endplate concavity (VEC) along the cage, which can be observed immediately after surgery with the previous PEEK cages. Hence, we hypothesized that this new cage has a lower risk of endplate injury at the time of insertion and prevents subsequent cage subsidence. The present study aimed to use VEC imaging findings to compare the local correction and VEC between patients who received the 3DTi cage with the findings in patients who received the conventional squared PEEK (sPEEK) cage.

## 2. Materials and Methods

### 2.1. Patient Population

Between January 2018 and June 2022, 58 consecutive patients who underwent anterior and posterior combined surgery with XLIF and posterior fusion (bilateral pedicle screw-rod system) for lumbar degenerative disease or adult spinal deformity were retrospectively reviewed. All clinical and radiological interventions were routine evaluations.

**Figure 2:**
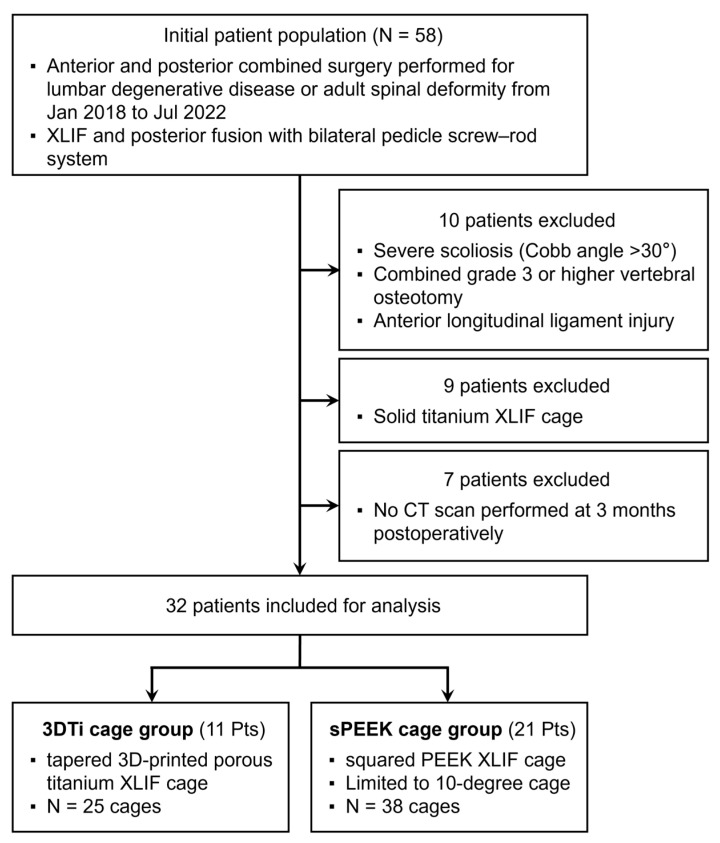
Flowchart for patients’ selection.

Patients with severe scoliosis (Cobb angle > 30°), those who underwent concomitant grade ≥ 3 osteotomies [12], and those with intraoperative injury to the anterior longitudinal ligament were excluded. In addition, nine patients with solid titanium cages were excluded. For postoperative evaluation, computed tomography (CT) was performed at 1 week and 3 months postoperatively; seven patients who had not undergone CT scans at 3 months were excluded. Finally, a total of 32 patients (mean age 74.1 ± 6.7 years; 22 females and 10 males) were analyzed in this study (Figure 2).

### 2.2. Surgical Procedure

XLIF was performed in a standardized manner using a one-incision technique, an NVM5 neuromonitoring device (NuVasive, San Diego, CA, USA), and a MaXcess retractor (NuVasive) [1] by spinal surgeons with sufficient lateral spine surgical experience. The patient was taped to the operating table in the lateral position, and the patient’s position and the operating table were adjusted so that the fluoroscopic equipment could obtain adequate images. XLIF was performed through an approximately 4 cm skin incision under fluoroscopic guidance, the MaXcess retractor was placed, and stepwise cage trials were performed to release and lift the interbody and determine the height of the cage. Then, the disc was curettaged and the cage inserted.

Posterior fusion surgery was performed the same day or 1 week later depending on the disease and surgical strategy. For corrective surgery for adult spinal deformity, a 1-week interval was allowed before posterior surgery because a two-stage procedure was planned to reduce surgical invasiveness. Posterior surgery was performed on the same day for other conditions. Patients with adequate alignment underwent posterior surgery with a percutaneous pedicle screw-rod system. Patients who needed more alignment correction or laminectomies for decompression were indicated for posterior fusion using the open technique. In such cases, bilateral facet joints were resected as required, and grade 1 or 2 osteotomy [12] was performed. Postoperatively, patients wore a hard corset for 3 months.

**Figure 3:**
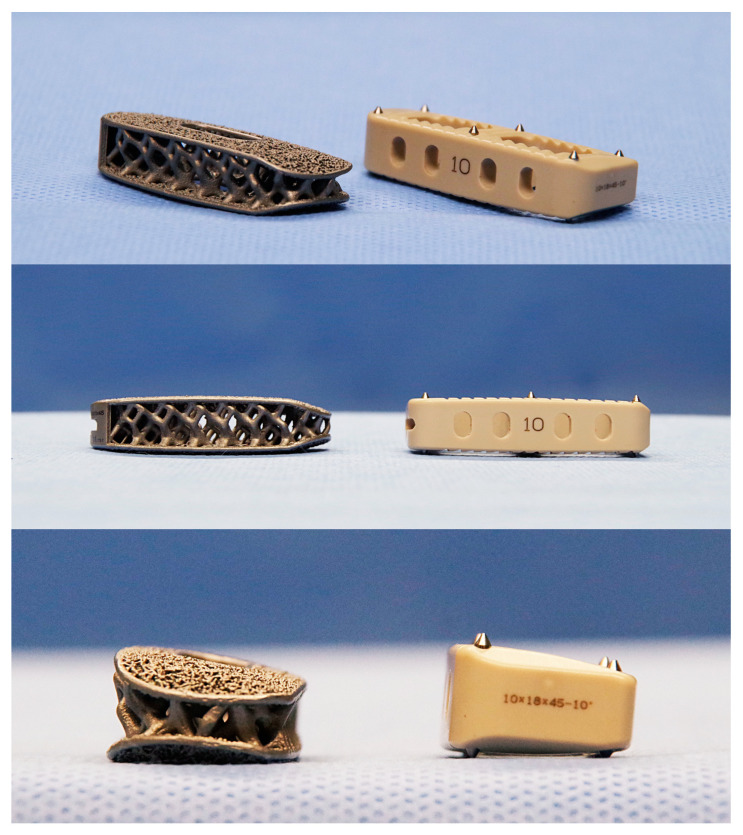
Appearance of 3DTi and sPEEK cages. The 3DTi cages are bullet-shaped and have smooth surfaces. The sPEEK cage has angular corners, spikes, and serrations.

During the study period, cages made of tapered 3D porous titanium (3DTi, Modulus; NuVasive) were used in 11 patients after February 2022, and cages made of sPEEK (CoRoent XL PEEK; NuVasive) were used in 21 patients until January 2022 (Figure 3). A custom-made hydroxyapatite mass was inserted into the 3DTi cage, whereas the PEEK cage was filled with grafting bone. The XLIF technique was identical regardless of cage type.

### 2.3. Patients’ Demographic and Operative Data

Age, sex, body mass index, comorbidities, and preoperative bone mineral density [BMD of the proximal femur measured using dual-energy X-ray absorptiometry (Prodigy; GE Healthcare, Chicago, IL, USA)] were recorded. The levels and number of intervertebral segments for which XLIF was performed, the property of each cage (height and angle), and postoperative complications were recorded. Only products with 10° angle 3DTi cages were used, so the intervertebral segments with sPEEK cages having angles > 10° were excluded from the analysis.

### 2.4. Radiological Assessments

**Figure 4:**
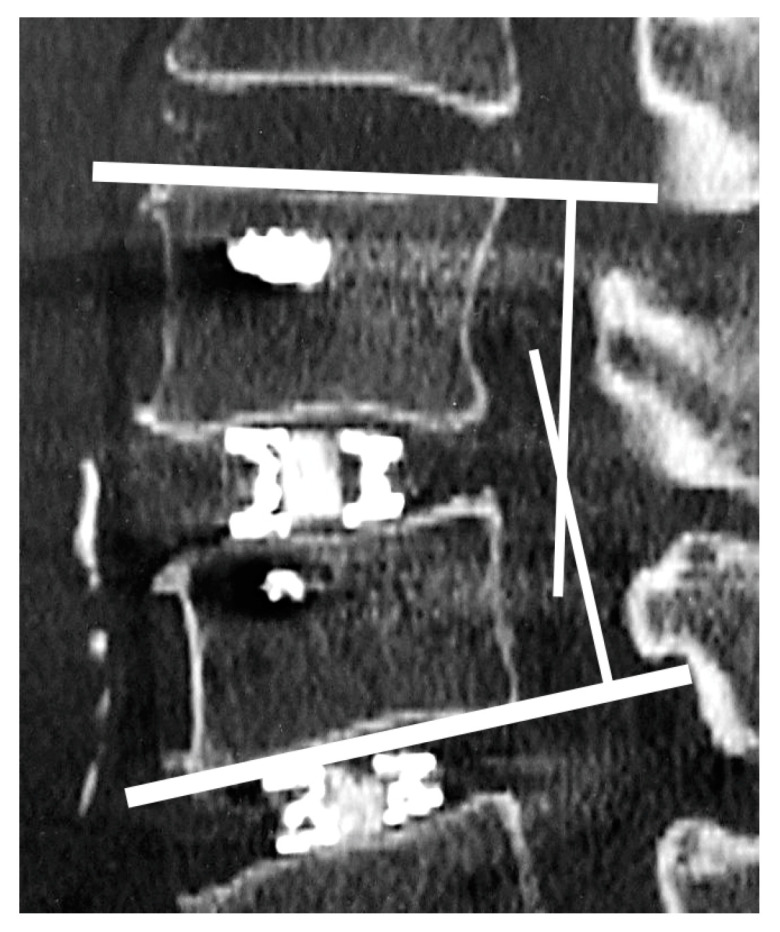
Intervertebral lordotic angle was defined as the angle formed by the cephalad endplate of the upper vertebra and the caudal endplate of the lower vertebra for each level.

X-ray images were obtained in the standard standing position. CT images were taken using standard methods (Aquilion ONE Nature Edition, Canon, Tokyo, Japan; tube voltage 135 kV), and multiplanar reconstruction (MPR) images were produced. Postoperative CT images of patients who underwent staged surgeries were taken after both scheduled surgeries were completed. For spinal alignment, lumbar scoliosis (Cobb angle), pelvic incidence (PI), lumbar lordosis (LL), pelvic tilt (PT), and sagittal vertical axis (SVA) were measured. Each intervertebral lordotic angle was measured preoperatively and at 1 week and 3 months postoperatively by CT-MPR sagittal imaging. The intervertebral lordotic angle was defined for a positive value for lordosis (Figure 4).

### 2.5. Vertebral Endplate Concavity (VEC) and Cage Subsidence

**Figure 5:**
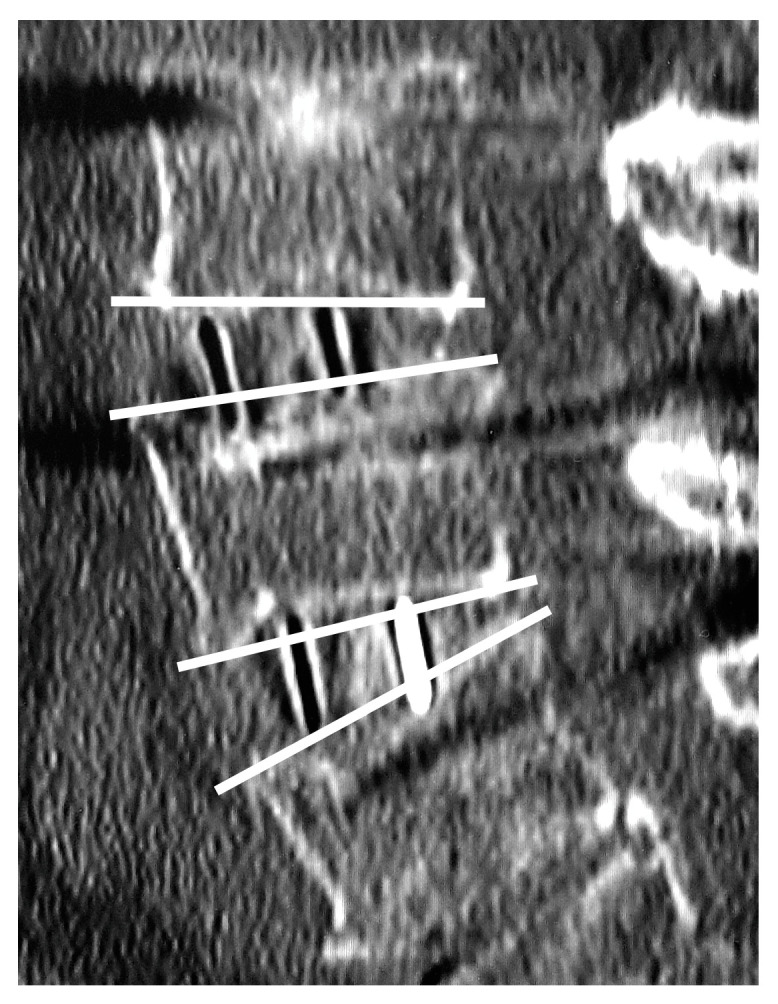
Vertebral endplate concavity (VEC) and cage subsidence. Both cages exceeded the end plates by ≥1 mm (VEC-positive) with Marchi classification grade 0.

VEC was defined as a concavity ≥ 1 mm along the cage or an obvious fracture of the vertebral endplate on first postoperative CT images compared with preoperative CT images. Additionally, VEC was investigated for any increase or change in CT images at three months postoperatively. The Marchi classification was used to assess cage subsidence [13] by comparing images immediately postoperatively and three months later on the following scale: grade 0 = 0–24% loss of postoperative disc height, grade I = 25–49%, grade II = 50–74%, and grade III = 75–100% (Figure 5).

### 2.6. Statistical Analysis

Data are presented as the mean and standard deviation for continuous variables and as number and percentage for categorical data. Statistical analyses using the Wilcoxon rank sum test, Fisher’s exact test, and Pearson’s Chi-squared test were performed in R version 4.2.1 (http://www.R-project.org (accessed on 1 July 2022)). Values of *p* < 0.05 were considered to be indicative of statistically significant differences.

## 3. Results

Patient demographic data showed no statistically significant differences: 11 patients in the 3DTi group averaged 74.7 years old, included 6 females, and had an average BMD of 0.692 g/cm^2^, and 21 patients in the sPEEK group averaged 73.8 years old, included 16 females, and had an average BMD of 0.693 g/cm^2^. There were no significant differences in the preoperative and 3-month postoperative alignments between the groups. The Cobb angles in the lumbar region were 12.9° in the 3DTi group and 12.7° in the sPEEK group, PI minus LL values were 36.3° vs. 25.9°, and SVA values were 114.3 mm vs. 76.0 mm, respectively (Table 1).

XLIF was performed from one to a maximum of four levels. All patients underwent combined posterior spinal fusion, and the range of fusion is shown in Table 2. XLIF-related complications included thigh symptoms in one patient in the 3DTi group and two in the sPEEK group, both of which were transient.

For a 10° angled cage, the 3DTi cage was used for 25 levels and the sPEEK cage for 38 levels (Table 3). Cage heights were >10 mm at 4 levels (16%) for the 3DTi cage vs. 13 levels (34%) for the sPEEK cage (*p* = 0.11). Preoperative local lordotic angles at each level were 4.3° for 3DTi vs. 4.7° for sPEEK (*p* = 0.90), which corrected to 12.3° vs. 9.1° (*p* = 0.029), respectively. At 3 months postoperatively, the local lordotic angles were 11.6° for 3DTi vs. 8.2° for sPEEK (*p* = 0.013).

**Figure 6:**
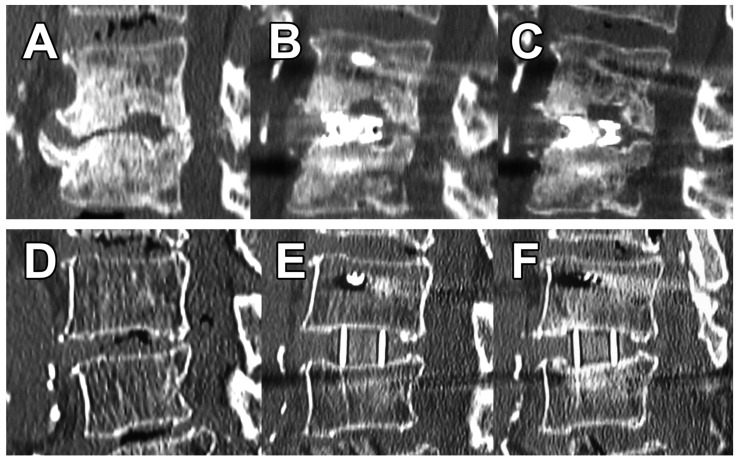
Differences in postoperative changes depending on cage. (**A**), preoperative degenerated interbody; (**B**), postoperative VEC caused by the 3DTi cage; (**C**), no further subsidence after 3 months. (**D**), preoperative; (**E**), postoperative VEC caused by the sPEEK cage; (**F**), cage subsidence and decreased lordosis (11° to 6°) after three months.

VEC was present in 2 (8.0%) levels for 3DTi vs. 17 (45%) levels for sPEEK (*p* = 0.002). There were no obvious fractures in either group. At 3 months postoperatively, no levels in the 3DTi group showed VEC progression (Figure 6A–C); however, eight (21%) levels in the sPEEK group showed VEC progression (Figure 6D–F) (*p* = 0.019). As a result, no levels in the 3DTi group and two (5.3%) levels in the sPEEK group were Marchi classification grade I. Grades II or III were not present in either group. There was also no cage dropout in either group.

## 4. Discussion

The study results showed that intraoperative endplate concavity was significantly less for XLIF using the novel tapered 3D-printed porous titanium cage than using the conventional sPEEK cage. In addition, further cage subsidence at 3 months was only seen using a sPEEK cage. This cage subsidence did not significantly affect spinal alignment at 3 months; however, there were significant differences between cages in the correction and maintenance of the local lordotic angle, with the novel titanium cages being superior. This finding was probably because the novel cages do not allow progressive cage subsidence.

First, only few studies have focused on the intraoperative endplate injuries (such as VEC). Satake et al. [4] evaluated the intraoperative endplate injuries by comparing the radiographs of patients with conventional cages before and after undergoing the surgery; they found that intraoperative endplate injuries occurred in 10.4% of patients, indicating that BMD and cage height are risk factors for such injuries. However, there were no significant differences in BMD or cage height between the two groups in our study, which suggested that other factors may have influenced the results. Adl Amini et al. [9] compared patients with 3DTi or sPEEK cages and evaluated cage subsidence using radiographs or CT imaging within 6 months after surgery (mean duration, 29.5 weeks). The researchers found that 3DTi cages had significantly less subsidence (Marchi grade 0 was 88.1% and 64.7% for 3DTi and sPEEK, respectively), and multivariate analysis showed that the use of the 3DTi cages was associated with significantly less subsidence. Although we avoided performing multivariate analysis due to the relatively small number of patients, a more short-term and detailed evaluation using CT imaging of all patients showed that the 3DTi cage was advantageous in preventing VEC and cage subsidence from immediately after surgery.

The frequent presence of VEC along the cage in images immediately after XLIF was a clinical question we wished to investigate in the present study. In some patients, the concavity gradually deepened, leading to poor alignment. Although we were uncertain if the dents were due to endplate preparation or cage insertion [2], our suspicion was that the cage was the cause of the unexpected VECs because VECs were observed even if there were no intraoperative problems with the endplate preparation. Our hypothesis was that problems with the endplate may be caused by the cage, leading to subsequent cage subsidence in some cases.

With respect to the underlying cause of VEC, friction generated during cage insertion is one possible cause. The surface of the trial is smooth, but that of the cage is uneven. For titanium-coated PEEK cages, the frictional forces generated during cage insertion are sufficient to strip the titanium coating [14]. In contrast, friction tests between a cancellous bone and a porous surface metal have shown that the friction curve is nonlinear [15,16], resulting in frictional stresses that cannot be analyzed simply. However, expulsion resistance tests to assess the risk of implant expulsion found that the surface structure had the greatest effect on resistance for postinsertion cages [6]. Therefore, it is possible that the cage surface, rather than differences in materials, may affect VEC occurrence. The sPEEK cage surface is marked with spikes and serrations. In contrast, the microstructure of the 3DTi surface that promotes bone ongrowth and ingrowth is on the order of hundreds of micrometers, so the surface of the 3DTi is smooth and may not pose a risk for VEC during insertion.

Second, the cage material should be considered as a factor that could have influenced the results of the present study. Titanium is an excellent metal to use in the living body owing to its strength and biocompatibility, and it has a long history of being used as an implant since the 1960s [17]. In contrast, the potential of metal ions to cause toxicity is still an important issue in orthopedic implants [18]. Using titanium alloys, which have excellent biocompatibility and corrosion resistance, can lower the toxicity of metal ions to the nervous, digestive, and immune systems [19] more than those caused by the other metal-based materials [20]. However, as titanium is usually much harder than the natural bones, the difference in the elastic modulus can cause failure [21]. One solution to this problem is to use porous metals.

Porous metal structures designed to mimic human bone tissue can be used as trabecular bone substitutes, because they have similar properties as the trabecular bone [22,23]. Moreover, they can be used to counteract the problem of biomechanical mismatch between the metallic implants and bone tissue [24]. To prevent bone resorption and promote favorable bone remodeling, the Young’s modulus of the implant should be similar to that of the bones (10–30 GPa) [25]. Additionally, the flow of bone marrow through the porous structure stimulates the bone-remodeling process [19]. Thus, porous metals have ideal properties for being used as implants.

Furthermore, the shape of the cage must be considered as a factor related to VEC. The effect on the vertebral endplate during cage insertion may be because of the cage geometry itself. The sPEEK cage has a near-rectangular geometry with angular corners that can clearly cause higher local stresses than what occurs with the 3DTi cage. In contrast, the 3DTi has a tapered tip and is more like a so-called “bullet shape” overall. Once lifted by the trial, the intervertebral space is narrowed again while waiting for the cage insertion. The cage inserted into the gap would be less likely to cause VEC with a more tapered than rectangular geometry. However, this hypothesis has not been confirmed in practice. Attempts should be made to focus on the specific timing of endplate damage and to minimize its occurrence [2].

Additionally, the cage angle was standardized to 10° in the current study, but there were variations in cage height. The 3DTi cages only exist in the 10° product, so the angle needed to be standardized. Cage height may influence endplate injury [4]. In contrast, another report found no association between early postoperative endplate injury and cage height [26]. Although the cage heights varied in in the current study, the bias was not significant. Furthermore, narrowing the analysis to only cage heights < 10 mm gave the same results (data not shown). For this reason, we did not standardize the cage height in this study.

Therefore, the clinical impact of cage evolution is of great interest to us; it is noteworthy that there were significant differences in the correction and maintenance of local angles between the 3DTi and sPEEK cages, suggesting that cage type may have an effect on intervertebral correction. It is highly likely that differences in intervertebral correction restoration by cage type affect global spinal alignment and clinical outcomes. However, it is also quite possible that the number of operated levels and the degree of VEC will determine whether or not there are statistically significant differences. The current study did not include only adult spinal deformity and the percentage of VEC was small; thus, the global spinal alignment might not be affected by the presence of VEC. Regarding clinical outcome, a previous report showed that although late cage subsidence worsened the rate of bony fusion, the clinical outcomes after cage subsidence observed in the early postoperative period did not significantly differ [26]. In contrast, another report showed that some levels of intraoperative endplate injury were associated with subsequently greater progressive cage subsidence than others and clinical improvement that was significantly greater in patients without intraoperative endplate injury than in those with endplate injury [3]. The reported results for early endplate abnormalities are not comparable because of the mixed timing of the observations. Our results support the natural notion that VEC is a risk for subsequent cage subsidence, and that local angles can worsen as a result. Since VEC is considered a risk factor for adverse clinical outcomes, we believe that 3DTi cages are useful for preventing those outcomes.

There were several study limitations that should be considered. First, this was not a randomized controlled trial. In addition, the surgical procedure and fusion range were based on the surgeon’s preference; however, cage selection was dictated solely by the timing of surgery and not by the surgeon’s choice. Second, the follow-up period was short, but this was because the 3DTi cage has only been available for a short period of time and the study design was intended to identify short-term changes. Furthermore, the study population was heterogeneous with adult spinal deformities requiring anterior–posterior combined corrective surgery and lumbar degenerative disease indicated for indirect decompression. In addition, we did not collect patient-reported outcomes. Finally, there may be measurement errors in assessing alignment, VEC, and cage subsidence on the radiographs and CT images.

In addition, although measurements were performed on a single model in the present study, it is not certain whether the present results apply when measurements are performed on other models because of intermodel errors. Generally, the possibility of a measurement error of <1 mm, depending on the model used, cannot be completely ruled out. However, in reference to past papers [4,26], the definition of VEC was more precisely defined as ≥1 mm in the current study. Despite these limitations, this comparison of imaging findings of the recently introduced new cage and a conventional cage showed significant differences and revealed some interesting characteristics of the cages.

## 5. Conclusions

The study results showed that the novel tapered 3D-printed porous titanium cage, recently introduced for XLIF, provided superior local correction and significantly less intraoperative VEC than observed for the conventional sPEEK cage. Additionally, although further cage subsidence at 3 months was observed with the conventional sPEEK cage, subsidence was not observed with the tapered 3D-printed porous titanium cage, which may be because the new cage reduces the load on the endplate during cage insertion. The impact of this novel cage feature on long-term outcomes, such as bony fusion and whole spinal alignment, can be studied in the future.

## Figures and Tables

**Table 1:** Patients’ demographics.

	3DTi *N* = 11	SPEEK *N* = 21	*p*-Value
Age, years	74.7 (6.3)	73.8 (7.1)	0.65
Sex, female	6 (55%)	16 (76%)	0.25
BMD, g/cm^2^	0.692 (0.088)	0.693 (0.122)	>0.99
Surgery			>0.99
1-staged	7 (64%)	12 (57%)	
2-staged	4 (36%)	9 (43%)	
Spinal alignment, pre-op			
Lumbar Cobb angle (°)	12.9 (8.6)	12.7 (9.4)	0.90
PI (°)	50.3 (8.1)	49.7 (8.3)	0.96
LL (°)	14.0 (25.3)	23.8 (12.8)	0.34
PI-LL (°)	36.3 (23.5)	25.9 (10.5)	0.28
PT (°)	30.1 (13.5)	26.0 (8.0)	0.36
SVA (mm)	114.3 (79.6)	76.0 (52.8)	0.28
Spinal alignment, post-op			
LL (°)	32.7 (14.4)	41.6 (15.3)	0.34
PI-LL (°)	13.7 (9.9)	8.6 (8.9)	0.39
PT (°)	20.7 (8.2)	22.6 (5.4)	0.78
SVA (mm)	30.2 (40.5)	31.1 (25.0)	0.46

BMD, bone mineral density; PI, pelvic incidence; LL, lumbar lordosis; PT, pelvic tilt; SVA, sagittal vertical axis.

**Table 2:** Surgical summary.

	3DTi *N* = 11	SPEEK *N* = 21	*p*-Value
Number of XLIF segment			>0.99
1	1 (9.1%)	2 (9.5%)	
2	6 (55%)	10 (48%)	
3	4 (36%)	8 (38%)	
4	0 (0%)	1 (4.8%)	
UIV			0.54
Upper thoracic	2 (18%)	1 (4.8%)	
Lower thoracic	1 (9.1%)	2 (9.5%)	
Lumbar	8 (73%)	18 (86%)	
LIV			0.63
Lumbar	6 (55%)	15 (71%)	
Sacrum	2 (18%)	2 (9.5%)	
Pelvis	3 (27%)	4 (19%)	
XLIF complication			
Thigh symptom	1 (33%)	2 (22%)	>0.99

UIV, upper instrumented vertebra; LIV, lower instrumented vertebra.

**Table 3:** Summary of each interbody compared by cage.

	3DTi Cage *N* = 25	SPEEK Cage N = 38	*p*-Value
Cage height			0.11
<10 mm	21 (84%)	25 (66%)	
≥10 mm	4 (16%)	13 (34%)	
Cage angle			
10°	25 (100%)	38 (100%)	
Cage level			0.29
L1–2	0 (0%)	2 (5.3%)	
L2–3	5 (20%)	14 (37%)	
L3–4	10 (40%)	13 (34%)	
L4–5	10 (40%)	9 (24%)	
Interbody angle *			
Pre-op (°)	4.3 (7.4)	4.7 (6.5)	0.90
Post-op (°)	12.3 (6.0)	9.1 (5.5)	0.029
3 months (°)	11.6 (6.4)	8.2 (5.7)	0.013
VEC			
Post-op	2 (8.0%)	17 (45%)	0.002
3 months progression	0 (0%)	8 (21%)	0.019
Marchi classification			0.51
Grade 0	25 (100%)	36 (95%)	
Grade I	0 (0%)	2 (5.3%)	

VEC, vertebral endplate concavity, * Lordotic angle of each level.

## Data Availability

The data presented in this study are available on request from the corresponding author. The data are not publicly available due to privacy or ethical restrictions.
